# Frontline Nurses' Job Satisfaction and Missed Nursing Care in a COVID-19 Dedicated Hospital in China: A Cross-Sectional Study

**DOI:** 10.1155/2024/5918935

**Published:** 2024-02-27

**Authors:** Xia Zhang, Jing Zhou, Fang Chen, Jing Yang, Zhixia Jiang, David Hali De Jesus

**Affiliations:** ^1^Department of Critical Care Medicine, Department of Nursing, Affiliated Hospital of Zunyi Medical University, Zunyi, China; ^2^School of Nursing, Philippine Women's University, Manila, Philippines; ^3^Department of Nursing, The Second Affiliated Hospital of Zunyi Medical University, Zunyi, China; ^4^Guizhou Nursing Vocational College, Guiyang, China

## Abstract

This study examined the current situation and relationship between missed nursing care (MNC) and job satisfaction among frontline nurses in a hospital dedicated to treating COVID-19 patients in China. Many dedicated hospitals were constructed or refurbished to centrally manage patients with COVID-19. Most nurses and doctors in these hospitals were redeployed from other departments or hospitals. This may have compromised nursing quality and job satisfaction. The omission of nursing care is a critical factor in assessing nursing quality; therefore, focusing on both MNC and job satisfaction is essential. This cross-sectional study used convenience and snowball sampling techniques to recruit frontline nurses working in a hospital for treating COVID-19 patients from November to December 2022. The questionnaires used in this study included sociodemographic information, job satisfaction, and the MISSCARE survey. Differences in job satisfaction and MISSCARE scores among participants' demographic deviations were explored using the Mann–Whitney Z test (two groups) and the Kruskal–Wallis H test (three or more groups). The correlation between participants' job satisfaction and missed nursing actions was analysed using Spearman's correlation analysis. The analysis included 306 frontline nurses. Frontline nurses' job satisfaction was high, and their MNC was low. The highest MNC was “offer rehabilitation care and guidance to patients in need every day.” The most reported reasons for the MNC were “urgent patient situations.” In addition, the job satisfaction scale, MNC scores, and reasons for MNC scores showed statistically significant differences among participants' demographic variables. Moreover, this study identified a negative correlation between frontline nurses' job satisfaction and MNC. Frontline nurses' job satisfaction was high, and their MNC was low. Frontline nurses' demographics were shown to affect their job satisfaction, MNC, and reported reasons. Furthermore, participants' job satisfaction can influence the MNC. Tailored interventions aimed at maintaining low levels of MNC should consider frontline nurses' demographic characteristics and job satisfaction.

## 1. Introduction

Missed nursing care (MNC) refers to any aspect of required care that is omitted, either in part or in whole, or delayed [1]. Evidence has shown that missed nursing activities can negatively affect patient safety, nursing care quality, and nurses' job satisfaction [2, 3]. Prior studies indicate that the COVID-19 pandemic has challenged nurses' working patterns because of the additional workload and psychological challenges [4, 5]. The challenges associated with COVID-19 in traditional working patterns reduce nurses' efficiency and well-being and threaten nursing quality, patients' safety, and nurses' job satisfaction [6, 7]. Thus, it is pivotal to consider the MNC and job satisfaction for frontline nurses caring for patients with COVID-19.

Several scholars have investigated this issue. A systematic review of five studies revealed higher MNC incidents among COVID-19 patients during the initial wave, which reduced in the second wave compared to incidents involving non-COVID-19 patients; these studies had contrasting findings, with some supporting and others contrasting the findings related to COVID-19 patients [8]. Two studies conducted in Sweden [9, 10] found that most nurses perceived the pandemic as having had a significant impact on the critical care workforce but less influence on MNC. A survey conducted in the Philippines [11] reported similar results, with MNC occurring at low levels. In Iran [12], Hosseini et al. (2022) reported that supportive and necessary care, such as “emotional support to the patient and/or family” and “feeding the patient when the food is still warm,” were missed more than any other form of care during the pandemic.

As mentioned previously, missed nursing activities can influence nurses' job satisfaction. Evidence suggests that the adequacy of personal protective equipment, nurse staffing levels, and patient safety culture can predict nurses' satisfaction during COVID-19 [11, 13]. Lavoie-Tremblay et al. (2022) found that nurses caring for COVID-19 patients experienced high chronic fatigue, poor quality of care, lower work satisfaction, and a higher intention to leave their organisation [14]. By contrast, Giménez-Espert et al. (2020) found that frontline nurses' satisfaction level was high [15].

These diverse results indicate that medical systems and policies vary among countries. For example, China suggested that COVID-19 patients should be collectively treated to contain the spread of the virus efficiently [16]. Consequently, many dedicated hospitals were promptly built or rebuilt [17]. As most dedicated hospitals did not have workers, healthcare workers from other hospitals or provinces were deployed [18]. To prevent these workers from becoming potential sources of infection, they were required to remain on-site until new workers arrived or until they completed their treatment duty [19]. Furthermore, they had to test negative for the disease at the end of the quarantine period before returning home. Unfamiliar work environments, dynamically changing personnel, exposure to the disease, a lack of experience in their new positions, and isolation from family members may have posed significant challenges to their work quality [11, 20]. Owing to these different national conditions, few studies have examined frontline nurses' job satisfaction and MNC in hospitals dedicated to COVID-19 in China. Researchers have postulated that there may be differences between China and other countries.

This study aimed to analyse the status and relationship between job satisfaction and MNC among frontline nurses in a hospital dedicated to COVID-19 in China. The findings of this study provide valuable insights for nursing managers and policymakers in the future.

## 2. Materials and Methods

### 2.1. Study Design and Settings

This cross-sectional study was conducted anonymously in a hospital dedicated to COVID-19 patients in China from November to December 2022, using convenience and snowball sampling techniques. This study was approved by the institutional review board.

### 2.2. Participants

All nurses deployed to Jiangjunshan Hospital in Guizhou Province to care for COVID-19 patients were eligible for this anonymous cross-sectional study. The inclusion criteria were that the nurses should have worked in the hospital for at least one month, should have provided nursing care directly to COVID-19 patients, could read and fill out a questionnaire, and had volunteered to participate. The exclusion criteria included not completing the questionnaire on time or taking less than 180 seconds (a pilot test in which 30 participants completed the questionnaire showed that reading and completing the questionnaire carefully took a minimum of 180 seconds).

The sample size was calculated using a method introduced by Wang and Ji (2020) and MNC proportions during the COVID-19 pandemic by Falk et al. (2022) [9, 21], considering a 15% invalid questionnaire rate. Therefore, the final sample size was determined as 284.

### 2.3. Measures

#### 2.3.1. Participants' Demographic Profiles

The researchers created the demographic profiles questionnaire (including gender, age, educational background, marital status, number of children, family support, friends support, professional titles, original hospital level, original hospital type, working departments in their original hospital and the dedicated hospital, position in their hospital, serving years, working days in the dedicated hospital, working hours per shift and week in the dedicated hospital, and time spent working in the dedicated hospital).

#### 2.3.2. Participants' Job Satisfaction

Frontline nurses' job satisfaction was measured using the Chinese version of the job satisfaction scale translated by Kachie Tetgoum (2021) [22] and developed by Paek et al. (2015) [23]. The scale comprised five items. Each item is answered on a 7-point Likert scale ranging from 1 (extremely dissatisfied) to 7 (extremely satisfied). To present the results better, the researchers divided the job satisfaction results into three levels: low (5–15), medium (16–25), and high (26–35), based on the total score of the questionnaire.

#### 2.3.3. Participants' MISSCARE Survey

The third section analysed the MNC and the reasons, using a Chinese version of the MISSCARE Survey from Si (2019) [24]; the original was created by Kalisch and Williams (2009) [1]. The survey had two parts: Part A (nursing care actions) included 24 items, and Part B (reasons for missed care) contained 19 items. The MNC items were answered on a 5-point Likert scale ranging from “always missed” to “never missed.” Another 19 items on the reasons for MNC were based on a 4-point Likert scale ranging from “significant reason” to “not a reason for MNC.”

To better present the results, the researchers divided the MNC results into three levels—low (24∼56), medium (57∼88), and high (89∼120)—based on the total score in section A. To treat all variables dichotomously, the researchers defined MNC in section A as reported “occasionally,” “frequently,” or “always,” similar to the MNC in the study by Falk et al. (2022) [9]. Furthermore, the researchers deemed the rate for each item of the MNC as high-incidence when its percentage was more significant than 50%, whereas those equal to or lower than 50% were low-incidence MNC.

The researchers also divided the reasons for the MNC survey into three levels—low (19∼37), medium (38∼57), and high (58∼76)—based on the total score in section B. In addition, the researchers adopted the method used in Falk et al. [9] to regard “significant” and “moderate” reasons as considered reasons for MNC. Furthermore, this study also deemed each item's occurrence rate as a high-incidence reason when its percentage was more significant than 50% and as a low-incidence reason when its ratio was equal to, or lower than, 50%.

#### 2.3.4. Reliability and Validity

According to the study by Kachie Tetgoum (2021) [22], the reliability of the Chinese version's Job satisfaction scale (Cronbach's alpha, *α*) was 0.88, indicating that this scale is a reliable tool for assessing nurses' job satisfaction.

Based on Si's (2019) results [24], the Chinese version of MISSCARE Survey's content validity index (CVI) for MNC and its reasons were 0.98 and 0.94, respectively, and their internal reliability (Cronbach's alpha, *α*) were 0.93 and 0.92, respectively, indicating that it is a reliable tool for assessing MNC.

Owing to the different participants in this and other studies, a pilot survey with 30 participants was conducted to test the reliability of the scales in this study. The participants' inclusion and exclusion criteria were the same as those of the survey participants mentioned above. The test results showed that the Job satisfaction scale's reliability (Cronbach's alpha, *α*) was 0.951 and the validity (Kaiser–Meyer–Olkin, KMO index) was 0.83. For the MNC questionnaire, the reliability (Cronbach's alpha, *α*) was 0.998 and its validity (KMO) was 0.835. Regarding the reasons for MNC questionnaire, the reliability and validity were 0.987 and 0.774, respectively, indicating that the questionnaires used in this study were reliable tools for this study.

#### 2.3.5. Data Collection

The data were collected between November and December 2022. The nursing staff in the research location was in workgroups on social media (WeChat) when they were deployed to the dedicated hospital. These workgroups have not yet been dissolved. First, the researchers posted a letter to the workgroups introducing the survey's aim, content, and instructions. Furthermore, the researchers emphasised that each nurse would participate in the study voluntarily and anonymously. Second, the final questionnaires were imported into a survey website (https://www.wjx.cn/), and the questionnaire link was shared with frontline nurses in the workgroups. Third, when respondents clicked on the link, they could see two options: “willing to participate” and “unwilling to participate.” Only participants who selected “willing to participate” could open the complete questionnaire. Thereafter, they could see an opening to the questionnaire introducing the aim, content, and instructions, and they participated in the survey through their accounts. In addition to frontline nurses who had left the workgroup, researchers contacted familiar frontline nurses who have worked at Jiangjunshan Hospital to recruit more nurses using a snowball sampling technique.

#### 2.3.6. Data Analysis

Software SPSS v22.0 (IBM Inc., Armonk, NY, USA) was used to analyse the data. Data were presented as frequencies, percentages, means (standard deviation, SD), and medians (IQRs). Skewness, kurtosis, and Q-Q plots were used to test data distribution. Considering the abnormal distribution of data in this study, differences in job satisfaction and MISSCARE scores among participants' demographic deviations were explored using the Mann–Whitney Z test (two groups) and Kruskal–Wallis H test (three or more groups). The correlation between participants' job satisfaction and missed nursing actions was analysed using Spearman's correlation analysis. For all analyses, *p* < 0.05 were considered statistically significant.

## 3. Results

### 3.1. Participants' Demographic Profiles

In total, 310 frontline nurses responded to the survey; however, six nurses took a short time to complete the questionnaire (less than 180 s). Therefore, 304 nurses were included in the statistical analysis. The results showed that 77.63% (236/304) of the participants were female. Nurses aged 31–40 years accounted for 56.58% (172/304), 90.13% (274/304) had a bachelor's degree, 79.93% (243/304) were married, and 44.08% (134/304) had only one child. The detailed demographic information is presented in Table 1.

### 3.2. Participants' Job Satisfaction

The results showed that most participants choose “often” or “always” as their answer for each item, and each item's median (IQR) score was from 6 (1) to 7 (1). Participants' total median (IQR) score of their job satisfaction in the dedicated hospital was 32 (5.75), a high-level score (i.e., 26∼35), indicating that the participants' satisfaction with each item was high. The details are listed in Table 2.

### 3.3. Participants' Missed Nursing Action

The results from this study demonstrated that the percentage of MNC items varied from 17.43% (53/304) to 27.96% (85/304), indicating that each MNC provided low-incidence care (i.e., lower than 50%). In addition, item 1 had the highest score, and its median (IQR) was 2 (2); other items scored a median (IQR) of 1 (1); and the total median (IQR) score of the questionnaire was 32 (22), a low-level score (i.e., 24–56). The three significant missed nursing activities in this study were “offer rehabilitation care and guidance to patients in need every day;” “emotional support for patient and/or family;” and “patient teaching about illness, tests, and diagnostic studies.” Additional information is presented in Table 3.

### 3.4. Participants' Reasons for Missed Nursing Care

This study found that the rate of each reason for missed care ranged from 18.09% (55/304) to 55.26% (168/304) and the median (IQR) for each item ranged from 1 (1) to 3 (2), with a total median (IQR) score of 37 (19), indicating that these were low-level reasons. However, the percentages of “urgent patient situations,” “unexpected rise in patient volume and/or acuity on the unit,” and “the nurse did no nursing work” items were greater than 50%, indicating that these three were high-incidence reasons for the MNC in this study. Other details are listed in Table 4.

### 3.5. Comparison between Job Satisfaction and the MISSCARE Scores among Participants' Demographic Characteristics

There were significant differences in frontline nurses' total job satisfaction scores by age (*p*=0.001), marital status (*p*=0.022), number of children (*p*=0.006), professional title (*p*=0.021), and original department (*p*=0.002). Higher job satisfaction was identified among frontline nurses who were older, married, had children, and higher professional titles, and were not originally from the ICU or emergency departments.

Regarding the MNC scores, significant statistical differences (*p* < 0.05) were observed among participants of different ages, family support, friend support, hospital types, and original department. Nurses with sufficient social support and older nurses reported fewer MNC. Nurses from the emergency department scored higher MNC points than those from other departments.

In addition, the study found significant differences (*p* < 0.05) in the reported reasons scores among frontline nurses based on various demographic and work-related factors. These factors include gender, age, family support, friend support, original hospital type, original department, time spent working in the dedicated hospital, working department in the dedicated hospital, and working hours per shift in the dedicated hospital. Frontline nurses who were male, younger, lacked support from family and friends, worked in comprehensive hospitals, originally worked in the ICU and emergency departments, worked in the dedicated hospital's ICU, and worked four to six hours per shift reported higher reasons scores. Detailed information is provided in Table 5.

### 3.6. The Correlations (Spearman) between the Respondents' Job Satisfaction and Their Missed Nursing Care Scores

The researchers used the total score of the MISSCARE A questionnaire as the dependent variable and the total score of the job satisfaction questionnaire, as well as each item's score of the job satisfaction questionnaire, as independent variables. Spearman's test was used to analyse the correlation because the data had an abnormal distribution. The results revealed a negative correlation between participants' job satisfaction and missed care action scores. In addition, each item in the job satisfaction questionnaire was negatively correlated with missed nursing actions (Table 6). These findings suggest that frontline nurses with higher job satisfaction tend to miss fewer nursing care actions.

## 4. Discussion

Both the quality of nursing care and nurses' job satisfaction has been crucial issues in healthcare systems. MNC is an essential quality indicator in clinical practice that can influence nurses' job satisfaction. Therefore, this study analysed the current situation of MNC and job satisfaction among frontline nurses in a dedicated hospital in China. Based on the results, frontline nurses' job satisfaction was high while their MNC was low. Job satisfaction among participants can still influence MNC, ultimately affecting the quality of nursing care provided.

### 4.1. Frontline Nurses' Job Satisfaction

This study's results showed that frontline nurses in the dedicated hospital had high job satisfaction levels. These findings suggest that frontline nurses were satisfied with their work environment in the dedicated hospital. However, these results differ from those of other studies exploring frontline nurses' job satisfaction during the COVID-19 crisis. Wang et al. (2022) reported that frontline nurses in Wuhan experienced moderate levels of compassion satisfaction during the first wave of the pandemic in Wuhan [25]. By contrast, our study was conducted at the end of 2022, the third year of the pandemic, when healthcare workers had gained more experience and skills and could adequately prepare for and cope with the pandemic. Giménez-Espert et al. (2020) found that frontline nurses' satisfaction was high [15], whereas Falk et al. (2022) reported that nurses' job satisfaction improved as the pandemic progressed [9]. Frontline nurses' job satisfaction was higher in the second wave than in the first. This study confirms the reasons for the differences between the two studies.

### 4.2. Frontline Nurses' Missed Nursing Care

This study's results demonstrate that the frontline nurses' MNC was low. Von Vogelsang et al. (2021) reported the presence of MNC during the COVID-19 pandemic [10]. However, their results showed a higher percentage of MNC compared to that shown in this study, such as the two items' occurrence percentages being more significant than 50% in their research. By contrast, the highest incidence rate in this study was only 27.96%. A study in Iran by Hosseini et al. (2022) [12] reported that “emotional support for patient and/or family,” “feeding patient when the food is still warm,” and “patient teaching about illness, tests, and diagnostic studies” were frequently missed items during the pandemic. Falk et al. (2022) also examined missed nursing during the first (November 2020) and second waves (May 2021) [9], finding that some items occurred more, while others occurred less, than did those in this study. In their research, the occurrence of “feeding patient when the food is still warm” and “setting up meals for the patient who feeds themselves” was 71.4% in the first wave and 79.4% in the second wave, respectively. The inconsistent results among these studies may be attributed to different survey times, places, and participants. A systematic review revealed a heightened incidence of MNC among COVID-19 patients during the initial wave and a diminished occurrence in comparison with non-COVID-19 patients in the second wave [8], which conformed to our postulation.

### 4.3. Frontline Nurses' Reported Reasons for Missed Nursing Care

Results from this study indicate that most reasons were low-incidence reasons, except for the “urgent patient situations” and “unexpected rise in patient volume and/or acuity on the unit” items. VonVogelsang et al. (2021) reported that the highest reasons for MNC were “unexpected rise in patient volume and/or acuity on the unit,” “urgent patient situations,” and “inadequate number of staff,” and their rates were from 32.4% to 79.8% in the first pandemic wave (May- June 2020) [10]. Falk et al. (2022) concluded that the most reported reasons for MNC in all samples were “inadequate staffing,” “urgent situations,” and “a rise in patient volume,” with rates ranging from 5.3% to 97.4% in the first wave and from 2.9% to 93.2% in the second wave [9]. Hosseini et al. (2022) reported that the significant reasons were “inadequate staff,” “urgent patient situations” (e.g., worsening of a patient's condition), and “unbalanced patient assignments.” Perhaps the different survey times, research locations, and participants could explain the inconsistent results among the three studies. For example, this survey was conducted from November to December 2022. Managers and administrators had prepared more adequately for the pandemic, and the supporting strategies and available supplies had improved. Labrague et al. (2022) found that nurse staffing levels and patient safety culture could predict MNC, confirming this study's findings [11]. In addition, the high-incidence reasons indicate that when staffing and scheduling frontline nurses, nursing managers should adopt a flexible schedule to meet urgent clinical demands and determine frontline nurses' working scope to avoid nurses doing no nursing work.

### 4.4. Comparison between Job Satisfaction and MISSCARE Scores among Participants' Demographic Characteristics

First, this study's results indicated that frontline nurses who were older, married, had children, held higher professional titles, and were not originally from the ICU or emergency department showed higher job satisfaction. Generally, nurses with these characteristics receive more support from their families and original hospitals. Zhang et al. (2020) found that older healthcare workers enjoyed better mental health during the COVID-19 pandemic [26], which corroborates the findings of this study. Nurses who originally worked in the ICU or emergency department showed lower job satisfaction, possibly because of their newly allocated units and high job requirements. When staffing and scheduling nurses, nursing managers tended to allocate nurses with experience in the ICU and emergency department to the ICU, where they faced critically ill patients. González-Gil et al. (2021) suggested that critical care and emergency nurses could be categorised as vulnerable populations owing to high workloads, high patient-nurse ratios, shift work, and deficiencies in communication [27]. These reasons may explain the present study's results. Therefore, nursing managers should focus on the needs and conditions of frontline nurses and offer targeted support and assistance.

Second, this study demonstrated that older participants who lacked adequate social support scored higher on the overall missed nursing action questionnaire. One potential reason for this could be that, as nurses' age grow, their experiences and knowledge increase, which could help them deal with job demands. As mentioned previously, older healthcare workers enjoy better mental health [26]. In addition, nurses with sufficient family and friend support had lower MNC scores, which may contribute to better social support. Nurses with such backing may also be more engaged in their work, resulting in less missed care. Thus, nursing managers should consider frontline nurses' age, family and social support, and their original departments to reduce MNC.

Third, this study found that nurses from comprehensive hospitals who were originally from the emergency department and intensive care unit had higher MNC scores. Nurses in specialised hospitals tend to have more experience in specific specialties, and when they move to a dedicated hospital, they can provide better care to patients in that area. However, nurses from comprehensive hospitals may face more choices surpassing their skill set, leading to a higher occurrence of MNC. Labrague et al. (2022) reported that hospital facility size could affect MNC [11], which is consistent with the findings of this study. Moreover, frontline nurses who worked in emergency departments and intensive care units were originally prone to being assigned to intensive care units in dedicated hospitals. Lobo et al. (2022) found that critical care nurses were disproportionately affected by the COVID-19 pandemic [28], and González-Gil et al. (2021) emphasised that critical care and emergency nurses can be categorised as vulnerable populations [27]. Thus, nursing managers should consider frontline nurses' original departments and hospital types to allocate nurses reasonably and thus maintain a low-level MNC.

Regarding the participants' serving years and gender, the results of Hosseini et al. (2022) showed that nurses with more than 10 years of work experience performed better than did those with less than 10 years of experience [12], which is consistent with the findings of this study. However, this study showed no statistically significant difference in MNC based on years of service. In addition, Hosseini et al. (2022) reported that the gender of nurses was significantly related to MNC (*p*=0.002), with MNC being significantly lower among male nurses than among female nurses. However, this relationship was not observed in the present study.

Furthermore, Hosseini et al.'s (2022) study found that 8-hour and 12-hour rotation shifts had the highest rate of missed necessary care, whereas 7-hour shifts had the lowest rate of MNC [12]. These results are inconsistent with this study's findings, probably due to the shorter shift duration in this study, whereby most frontline nurses (86.18%) reported working for 4–6 hours per shift. In summary, nursing managers should consider frontline nurses' age, family and friend support, hospital type, and original department to reduce MNC.

Finally, the survey results indicated higher scores for male and younger frontline nurses, those with inadequate support from family and friends, those working in comprehensive hospitals or the dedicated hospitals' ICU, and those who originally worked in the ICU and emergency department and had a 4–6 hours working shift. Zhang et al. (2020) highlighted the varying levels of distress and depression experienced by the different genders [26]. In addition, a lack of social support can negatively affect nurses' emotional states, particularly those working in comprehensive hospitals, ICU, and emergency departments. Furthermore, Liu et al. (2022) found that frontline nurses in severe isolation wards worked shorter shifts of four hours compared to those in fever clinics and observation wards, who worked for 6–8 hours [29]. However, nurses who worked in 2021 and 2022 may have reported higher scores than those who worked in 2020 because of memory deviation over time. Therefore, nursing managers should prioritise frontline nurses' work conditions to enhance nursing quality.

### 4.5. The Correlations between the Frontline Nurses' Job Satisfaction and Their MNC

This study found a negative correlation between job satisfaction and missed care. In addition, this study also indicated that nurses miss less nursing care when they are more satisfied with their jobs, fellow workers, supervisors, hospital policy, and hospital support. A study by Gurková et al. (2022) in Czech acute care hospitals during the COVID-19 pandemic identified that overtime work, nurses' perception of the “Nursing foundations for the quality of care,” and their satisfaction with their current position could predict the incidence of missed care [30]. Falk et al. (2022) determined that MNC could influence both patient outcomes and nurses' work environments [9], and that bedside nurses should develop quality indicators in critical care to address the reasons for MNC. In addition, Gurková et al. (2022) indicated that monitoring the conditions and aspects of the nurse work environment in hospitals and continuously considering nurses' concerns about the work environment on an ongoing basis are essential strategies for nurse supervision and for policymakers [30]. Labrague and de Los Santos (2021) pointed out that decreased job satisfaction could increase the fear of COVID-19 and chronic fatigue, and that resilience could reduce the effects of pandemic fatigue on clinical nurses' mental health, sleep quality, and job contentment [7]. This study confirmed the findings of the present study and indicated that hospital managers could improve nurses' job satisfaction by improving their nursing quality through their jobs, fellow workers, supervisors, hospital policies, and hospital support.

### 4.6. Limitations and Recommendation

The limitations of this study are as follows. First, as this study was conducted using a questionnaire survey, self-report bias is an evident limitation. Second, this study only included frontline nurses in one dedicated hospital, which may limit its generalizability to other locations. Finally, this study was conducted at the end of 2022, and frontline nurses who worked at dedicated hospitals in 2020 and 2021 may have experienced memory deviations over time. Thus, multicentre, large-sample, and more objective assessment studies are required.

Frontline nurses are an essential group that determines service quality during the response to crises relevant to emerging infectious diseases. Nursing managers should pay attention to the nurses' needs and provide tailored support and assistance based on age, gender, marital status, social support, and working environment. In addition, the results indicated that hospital managers could improve nurses' job satisfaction by improving their nursing quality through their jobs, fellow workers, supervisors, hospital policies, and hospital support.

## 5. Conclusion

Frontline nurses' demographics can affect their job satisfaction, MNC, and reported reasons. As the pandemic progressed, frontline nurses reported improvements in job satisfaction and a reduction in the number of MNCs. However, it should be noted that job satisfaction among participants could still influence MNC, ultimately affecting the quality of nursing care provided.

## 6. Implications for Nursing Management

The COVID-19 pandemic is highly contagious and poses challenges to nurses' work efficiency and emotional health. To reduce the occurrence of MNC and enhance nursing quality, hospital administrators and nursing managers should focus on improving nurses' job satisfaction by improving their work environments, supporting their relationships with colleagues and supervisors, implementing effective hospital policies, and providing adequate hospital support. Policymakers and nursing managers should thoroughly assess the factors influencing MNC, such as nurses' characteristics. To some extent, it is possible to maintain a low level of MNC under similar conditions in the future once policymakers and nursing managers use this information to make reasonable and feasible decisions or policies.

## Figures and Tables

**Table 1:** Participants' demographic characteristics.

Variables (*N* = 304)	Categories	Frequency	Percentage
Gender	Male	68	22.37
	Female	236	77.63

Age median (IQR)	32 (6)		

Age (year)	23∼30	107	35.20
	31∼40	172	56.58
	41∼53	25	8.22

Educational level	Junior college or below	25	8.22
	Bachelor's degree	274	90.13
	Master's degree or above	5	1.64

Marital status	Single	56	18.42
	Married	243	79.93
	Others	5	1.64

Children number	0	78	25.66
	1	134	44.08
	2	90	29.61
	3	2	0.66

Family support	Adequate	253	83.22
	Inadequate	51	16.78

Friend support	Adequate	252	82.89
	Inadequate	52	17.11

Professional title	Junior	172	56.58
	Intermediate	114	37.50
	Senior	18	5.92

Original hospital level	Tertiary	259	85.20
	Secondary	44	14.47
	Others	1	0.33

Original hospital type	Comprehensive hospital	283	93.09
	Specialized hospital	21	6.91

Original department	Intensive care unit	145	47.70
	Emergency department	88	28.95
	Outpatient department	3	0.99
	Surgery department	27	0.88
	Medicine department	25	8.22
	Others	16	5.26

Original post	Clinical nurse	235	77.30
	Head nurse	34	11.18
	Others	35	11.51

*Years of service median (IQR) 10 (5)*			
Years of service (year)	3∼10	173	56.91
	11∼20	110	36.18
	21∼30	21	6.91

Time of working in the dedicated hospital	2020	42	13.82
	2021	111	36.51
	2022	99	32.57
	Others (consecutive two or three years)	52	17.11

*Days of working in the dedicated hospital median (IQR)*			47 (51.75)
Days of working in the dedicated hospital	30∼60	207	68.09
	61 or above	97	31.91

Working department in the dedicated hospital	Intensive care unit	174	57.24
	Isolation ward	113	37.17
	Other departments	17	5.59

*Working hours per shift in the dedicated hospital median (IQR)*			6 (2)
Working hours per shift in the dedicated hospital (hours)	4∼6	262	86.18
	7∼8	31	10.20
	8.5 or above	11	3.62

*Working hours per week in the dedicated hospital median (IQR)*			36 (14)
Working hours per week in the dedicated hospital (hours)	21∼30	123	40.46
	31∼40	78	25.66
	41 or above	103	33.88

**Table 2:** Participants' job satisfaction.

Items	Never/1 point	Rarely/2 points	Seldom/3 points	Sometimes/4 points	Frequently/5 points	Often/6 points	Always/7 points	Scores median (IQR)
	*N* (%)	*N* (%)	*N* (%)	*N* (%)	*N* (%)	*N* (%)	*N* (%)	
(1) I am satisfied with my overall job	2 (0.66)	3 (0.99)	0 (0)	25 (8.22)	27 (8.88)	102 (33.55)	145 (47.70)	6 (1)
(2) I am satisfied with my fellow workers	2 (0.66)	1 (0.33)	1 (0.33)	26 (8.55)	20 (6.58)	99 (32.57)	155 (50.99)	7 (1)
(3) I am satisfied with my supervisor	3 (0.99)	4 (1.32)	2 (0.66)	30 (9.87)	15 (4.93)	89 (29.28)	161 (52.96)	7 (1)
(4) I am satisfied with the hospital's policy	4 (1.32)	1 (0.33)	11 (3.62)	31 (10.20)	21 (6.91)	87 (28.62)	149 (49.01)	6 (1)
(5) I am satisfied with the support provided by this hospital	4 (1.32)	1 (0.33)	7 (2.30)	33 (10.86)	19 (6.25)	82 (26.97)	158 (51.97)	7 (1)
Total								32 (5.75)

**Table 3:** Results of participants' missed nursing action (in descending order of each missed nursing action's percentage).

Items	Never/1 point	Rarely/2 points	Occasionally/3 points	Frequently/4 points	Always/5 points	Scores median (IQR)	Missed care	
	*N* (%)	*N* (%)	*N* (%)	*N* (%)	*N* (%)		Yes *N* (%)	Rank
(1) Offer rehabilitation care and guidance to patients in need every day	139 (45.72)	80 (26.32)	38 (12.50)	15 (4.93)	32 (10.53)	2 (2)	85 (27.96)	1
(10) Emotional support for patient and/or family	150 (49.34)	85 (27.96)	24 (7.89)	15 (4.93)	30 (9.87)	1 (1)	69 (22.69)	2
(9) Patient teaching about illness, tests, and diagnostic studies	161 (52.96)	75 (24.67)	21 (6.91)	12 (3.95)	35 (11.51)	1 (1)	68 (22.37)	3
(3) Assess the patient's risk in time to offer foreseeable nursing	167 (54.93)	70 (23.03)	16 (5.26)	19 (6.25)	32 (10.53)	1 (1)	67 (22.04)	4
(14) Patient discharge planning and teaching	171 (56.25)	66 (21.71)	18 (5.92)	13 (4.28)	36 (11.84)	1 (1)	67 (22.04)	5
(4) Assist patient with dining and dietary education	173 (56.91)	64 (21.05)	17 (5.59)	22 (7.24)	28 (9.21)	1 (1)	67 (22.04)	6
(11) Patient bathing/skin care	184 (60.53)	54 (17.76)	14 (4.61)	15 (4.93)	37 (12.17)	1 (1)	66 (21.71)	7
(22) Know patient's condition well by checking their medical records	171 (56.25)	68 (22.37)	14 (4.61)	17 (5.59)	34 (11.18)	1 (1)	65 (21.38)	8
(2) Turning patient every 2 hours	174 (57.24)	66 (21.71)	19 (6.25)	15 (4.93)	30 (9.87)	1 (1)	64 (21.05)	9
(17) Focused reassessments according to patient condition	184 (60.53)	56 (18.42)	12 (3.95)	13 (4.28)	39 (12.83)	1 (1)	64 (21.05)	10
(21) Assess the effectiveness of medications	184 (60.53)	58 (19.08)	11 (3.62)	12 (3.95)	39 (12.83)	1 (1)	62 (20.39)	11
(19) Response to call light is initiated within 5 min	203 (66.78)	39 (12.83)	12 (3.95)	13 (4.28)	37 (12.17)	1 (1)	62 (20.39)	12
(8) Full documentation of all necessary data	192 (63.16)	50 (16.45)	12 (3.95)	13 (4.28)	37 (12.17)	1 (1)	62 (20.39)	13
(5) Medications administered within 30 minutes before or after scheduled time	190 (62.50)	53 (17.43)	11 (3.62)	12 (3.95)	38 (12.50)	1 (1)	61 (20.07)	14
(12) Mouth care	207 (68.09)	36 (11.84)	9 (2.96)	13 (4.28)	39 (12.83)	1 (1)	61 (20.07)	15
(24) Skin/wound care	193 (63.49)	51 (16.78)	9 (2.96)	13 (4.28)	38 (12.50)	1 (1)	60 (19.74)	16
(20) PRN medication requests acted on within 15 minutes	198 (65.13)	47 (15.46)	5 (1.64)	18 (5.92)	36 (11.84)	1 (1)	59 (19.41)	17
(23) Assist with toileting needs within 5 minutes of request	186 (61.18)	60 (19.74)	9 (2.96)	12 (3.95)	37 (12.17)	1 (1)	58 (19.08)	18
(13) Hand washing	213 (70.07)	33 (10.86)	5 (1.64)	11 (3.62)	42 (13.82)	1 (1)	58 (19.08)	19
(16) Make the rounds of the wards based on patient's grading nursing care standard each shift	219 (72.04)	28 (9.21)	5 (1.64)	7 (2.30)	45 (14.80)	1 (1)	57 (18.75)	20
(18) IV/central line site care and assessments according to hospital policy	204 (67.11)	44 (14.47)	5 (1.64)	13 (4.28)	38 (12.50)	1 (1)	56 (18.42)	21
(15) Bedside glucose monitoring as ordered	225 (74.01)	24 (7.89)	4 (1.32)	6 (1.97)	45 (14.80)	1 (1)	55 (18.09)	22
(7) Monitoring intake/output	234 (76.97)	15 (4.93)	7 (2.30)	6 (1.97)	42 (13.82)	1 (1)	55 (18.09)	23
(6) Vital signs assessed as ordered	225 (74.01)	26 (8.55)	4 (1.32)	8 (2.63)	41 (13.49)	1 (1)	53 (17.43)	24
Total						32 (22)		

**Table 4:** Participants' reasons for missed nursing care (in descending order of each missed nursing care's percentage).

Items	Not a reason/1 point	Minor reason/2 points	Moderate reason/3 points	Significant reason/4 points	Scores median (IQR)	Reasons for MNC	
	*N* (%)	*N* (%)	*N* (%)	*N* (%)		Yes *N*(%)	Rank
(2) Urgent patient situations (e.g., worsening of a patient's condition)	50 (16.45)	86 (28.29)	76 (25.00)	92 (30.26)	3 (2)	168 (55.26)	1
(3) Unexpected rise in patient volume and/or acuity on the unit	60 (19.74)	80 (26.32)	83 (27.30)	81 (26.64)	3 (2)	164 (53.95)	2
(4) Nurse did no nursing work	71 (23.36)	81 (26.64)	83 (27.30)	69 (22.70)	2.5 (1)	152 (50.00)	3
(1) Inadequate number of staff	80 (26.32)	77 (25.33)	82 (26.97)	65 (21.38)	2 (2)	147 (48.36)	4
(6) Medications were not available when needed	107 (35.20)	94 (30.92)	68 (22.37)	35 (11.51)	2 (2)	103 (33.88)	5
(8) Tension or communication breakdowns with patients	123 (40.46)	94 (30.92)	62 (20.39)	25 (8.22)	2 (2)	87 (28.62)	6
(5) Unbalanced patient assignments	126 (41.45)	93 (30.59)	57 (18.75)	28 (9.21)	2 (2)	85 (27.96)	7
(7) Inadequate hand-off from previous shift or sending unit	117 (38.49)	105 (34.54)	56 (18.42)	26 (8.55)	2 (2)	82 (26.97)	8
(9) Supplies/equipment not available when needed	138 (45.39)	89 (29.28)	48 (15.79)	29 (9.54)	2 (2)	77 (25.33)	9
(19) Underdeveloped management and quality assurance system	139 (45.72)	88 (28.95)	44 (14.47)	33 (10.86)	2 (2)	77 (25.33)	10
(12) Tension or communication breakdowns with other ancillary/support departments	149 (49.01)	82 (26.97)	48 (15.79)	25 (8.22)	2 (1)	73 (24.01)	11
(10) Supplies/equipment not functioning properly when needed	146 (48.03)	87 (28.62)	47 (15.46)	24 (7.89)	2 (1)	71 (23.36)	12
(16) Patient's and family's refusal	139 (45.72)	95 (31.25)	50 (16.45)	20 (6.58)	2 (1)	70 (23.03)	13
(11) Lack of backup support from team members	147 (48.36)	88 (28.95)	43 (14.14)	26 (8.55)	2 (1)	69 (22.70)	14
(18) Nurses with less job responsibility	144 (47.37)	91 (29.93)	41 (13.49)	28 (9.21)	2 (1)	69 (22.70)	15
(14) Tension or communication breakdowns within the medical staff	154 (50.66)	88 (28.95)	43 (14.14)	19 (6.25)	2 (1)	62 (20.39)	16
(15) Delayed communication between novices and nurses on patients' care	143 (47.04)	99 (32.57)	43 (14.14)	19 (6.25)	2 (1)	62 (20.39)	17
(17) Underdeveloped role description/workflow	145 (47.70)	101 (33.22)	37 (12.17)	21 (6.91)	2 (1)	58 (19.08)	18
(13) Tension or communication breakdowns within the nursing team	173 (56.91)	76 (25.00)	35 (11.51)	20 (6.58)	1 (1)	55 (18.09)	19
Total					37 (19)		

**Table 5:** Comparison between job satisfaction and MISSCARE scores among participants' demographic characteristics.

Variables (*N* = 304)	Categories	*N* (%)	Job satisfaction total scores			Missed nursing care total scores			Reasons for missed care total scores		
			Median (IQR)	Statistics	*p*	Median (IQR)	Statistics	*p*	Median (IQR)	Statistics	*p*
Gender	Male	68 (22.37)	31.50 (8)	−0.537	0.591	32.50 (75.5)	−1.504	0.133	38.5 (21.5)	−2.055	0.040^*∗*^
	Female	236 (77.63)	31 (7)			29.50 (21.8)			36 (18.7)		

Age (year)	23∼30	107 (35.20)	30 (8)	14,219	0.001^*∗∗*^	30.00 (23.0)	10.863	0.004^*∗∗*^	37 (21)	9.603	0.008^*∗∗*^
	31∼40	172 (56.58)	32 (6)			32.00 (23.8)			37.5 (20)		
	41∼53	25 (8.22)	34 (4)			25 (3.5)			30 (14)		

Educational level	Junior college or below	25 (8.22)	30 (9.50)	0.554	0.758	26 (11)	4.009	1.135	35 (29)	0.374	0.829
	Bachelor's degree	274 (90.13)	31 (7)			30 (22.3)			37 (19)		
	Master's degree or above	5 (1.64)	34 (6.5)			31 (55)			32 (25)		

Marital status	Single	56 (18.82)	30 (6)	7.665	0.022^*∗*^	30 (21.8)	0.629	0.73	35 (25)	0.064	0.969
	Married	243 (79.93)	32 (6)			30 (21)			37 (19)		
	Others	5 (1.64)	30 (9)			30 (48)			36 (7.5)		

Children number	0	78 (25.66)	30 (9)	12.465	0.006^*∗*^	30 (21)	4.276	0.233	36 (21.3)	1.452	0.693
	1	134 (44.08)	32 (6.2)			31 (25.3)			37 (20.3)		
	2	90 (29.61)	32.5 (5)			29 (18.3)			38 (17.5)		
	3	2 (0.66)	32.5 (5)			24 (0)			31 (0)		

Family support	Adequate	253 (83.22)	31 (7)	−0.122	0.903	28 (11.5)	−11.305	<0.001^*∗∗*^	35 (17)	−6.592	<0.001^*∗∗*^
	Inadequate	51 (16.78)	31 (7)			117 (17)			52 (31)		

Friend support	Adequate	252 (82.89)	31 (7)	−0.379	0.704	28 (11)	−11.391	<0.001^*∗∗*^	35 (17)	−6.683	<0.001^*∗∗*^
	Inadequate	52 (17.11)	30.5 (7.7)			116.5 (17)			52 (30.5)		

Professional title	Junior	172 (56.58)	30 (8)	7.707	0.021^*∗*^	30 (22)	1.323	0.516	37.5 (21)	5.205	0.074
	Intermediate	114 (37.50)	32 (6)			31 (21.3)			36.5 (18.3)		
	Senior	18 (5.92)	35 (5)			28 (10.3)			29.5 (12.8)		

Original hospital level	Tertiary	259 (85.20)	32 (7)	3.069	0216	31 (23)	2.678	0.262	36 (18)	1.004	0.605
	Secondary	44 (14.47)	30 (9.7)			28 (11.5)			39 (25)		
	Others	1 (0.33)	26 (0)			47 (0)			39 (0)		

Original hospital type	Comprehensive hospital	283 (93.09)	31 (8)	−1926	0.054	31 (23)	−3.071	0.002^*∗∗*^	37 (20)	−3.106	0.002^*∗∗*^
	Specialized hospital	21 (6.91)	34 (5)			24 (7)			29 (15.5)		

Original department	Intensive care unit	145 (47.70)	30 (8)	19.127	0.002^*∗∗*^	30 (29)	16.357	0.006^*∗∗*^	30 (22)	22.565	<0.001^*∗∗*^
	Emergency department	88 (28.95)	30 (7.7)			34.5 (19.8)			37.5 (15.8)		
	Outpatient department	3 (0.99)	35 (3)			24 (1)			21 (3)		
	Surgery department	27 (0.88)	35 (4)			24 (13)			27 (21)		
	Medicine department	25 (8.22)	33 (5)			25 (7.5)			29 (16.5)		
	Others	16 (5.26)	32 (5)			30 (73.3)			32.5 (21.7)		

Original post	Staff nurse	235 (77.30)	30 (8)	4.479	0.106	30 (22)	0.355	0.837	37 (21)	1.110	0.575
	Head nurse	34 (11.18)	34.5 (5)			30 (20.3)			34 (21.5)		
	Others	35 (11.51)	31 (7)			29 (21)			36 (22)		

Years of service (year)	3∼10	173 (56.91)	30 (8)	4.329	0.115	29 (22)	0.931	0.628	37 (20)	2.381	0.304
	11∼20	110 (36.18)	33 (7)			31.5 (24)			35 (10.2)		
	21∼30	21 (6.91)	31 (7)			29 (14)			39 (18.5)		

Time of working in the dedicated hospital	2020	42 (13.82)	34 (5)	6.605	0.086	27 (12)	3.613	0.306	27.5 (18)	13.765	0.003^*∗∗*^
	2021	111 (36.51)	30 (8)			31 (19)			39 (18)		
	2022	99 (32.57)	32 (8)			31 (25)			38 (20)		
	Others (consecutive two or three years)	52 (17.11)	32 (5)			32.5 (32.5)			35 (20.2)		

Days of working in the dedicated hospital	30∼60	207 (68.09)	30 (8)	−0.946	0.344	30 (22)	−0.466	0.641	38 (20)	−1.692	0.091
	61 or above	97 (31.91)	32 (7)			30 (21.5)			35 (18)		

Working department in the dedicated hospital	Intensive care unit	174 (57.24)	30 (8)	5.931	0.052	31.5 (22.3)	3.003	0.223	38 (19.3)	9.596	0.008^*∗∗*^
	Isolation ward	113 (37.17)	32 (5)			28 (19.5)			33 (18)		
	Other departments	17 (5.59)	32 (6)			34 (23)			39 (19.5)		

Working hours per shift in the dedicated hospital (hours)	4∼6	262 (86.18)	30 (8)	3.601	0.165	30 (23)	2.504	0.286	38 (20)	7.379	0.025^*∗*^
	7∼8	31 (10.20)	34 (5)			29 (16)			30 (17)		
	8.5 or above	11 (3.62)	34 (9)			26 (14)			35 (14)		

Working hours per week in the dedicated hospital (hours)	21∼30	123 (40.46)	30 (7)	0.059	0.971	31 (21)	1.188	0.552	37 (20)	0.916	0.633
	31∼40	78 (25.66)	32 (8)			29 (30.3)			35.5 (19.2)		
	41 or above	103 (33.88)	32 (7)			30 (21)			37 (192)		

*Note*: ^∗^*p* < 0.05, ^∗∗^*p* < 0.01.

**Table 6:** The correlations (Spearman) among participants' total scores and items of job satisfaction and their missed nursing cares.

Items	Missed nursing care scores	
	Coefficient	*P*
Total score of the job satisfaction	−0.337	<0.001^*∗∗*^
(1) I am satisfied with my overall job	−0.294	<0.001^*∗∗*^
(2) I am satisfied with my fellow workers	−0.280	<0.001^*∗∗*^
(3) I am satisfied with my supervisor	−0.276	<0.001^*∗∗*^
(4) I am satisfied with the hospital's policy	−0.314	<0.001^*∗∗*^
(5) I am satisfied with the support provided by this hospital	−0.298	<0.001^*∗∗*^

*Note.* ^*∗*^ *p* < 0.05^*∗∗*^*p* < 0.01.

## Data Availability

The data that support the findings of this study are available from the corresponding authors and the first author upon reasonable request.
